# OCD Symptoms and Capgras Syndrome in a 13-Year-Old Girl Following Right-Sided Brain Surgery

**DOI:** 10.1155/crps/8280525

**Published:** 2025-12-03

**Authors:** Carolyn Lucy Yoakum, Tasmima Tazin, Matthew J. Greve

**Affiliations:** ^1^Psychiatry Residency Program, National Capital Region, Walter Reed National Military Medical Center, 8901 Rockville Pike, Bethesda 20889, Maryland, USA; ^2^Defense Health Agency, Department of Behavioral Health, Child and Adolescent Psychiatry Fellowship , National Capital Region, Walter Reed National Military Medical Center, 8901 Rockville Pike, Bethesda 20889, Maryland, USA

## Abstract

This case report documents a rare presentation of obsessive–compulsive-like symptoms co-occurring with Capgras syndrome following surgical resection of a right-sided frontal lobe pleomorphic xanthoastrocytoma in a 13-year-old girl. The patient initially presented with headache and intractable vomiting, leading to the discovery of a right-sided frontal lobe tumor. Postoperatively, the patient exhibited cognitive and behavioral changes manifesting as misidentification beliefs regarding the identities of loved ones and associated distressing thoughts. Comprehensive psychiatric evaluation using validated assessment tools confirmed these presentations. This report contributes to understanding the complex interplay between neurological, psychiatric, and cognitive factors in the development of these rare postsurgical neuropsychiatric complications following frontal lobe tumor resection.

## 1. Introduction

Capgras syndrome is a rare neuropsychiatric misidentification syndrome where an individual perceives that a person, animal, or object familiar to them has been replaced by an identical imposter. Postsurgical neuropsychiatric complications, while uncommon, represent significant clinical challenges in pediatric neurosurgical patients. This case presents the development of Capgras syndrome with associated obsessive–compulsive-like symptoms in a young patient following right-sided frontal brain surgery.

## 2. Case Presentation

A 13-year-old girl, previously healthy, underwent neurosurgical intervention to remove a 6.4 cm × 6.1 cm × 6.3 cm pleomorphic xanthoastrocytoma located in the right frontal region after presenting to the emergency department with headache for several months and acute onset of vomiting. No focal neurological deficits were present, and the patient had no prior psychiatric or medical conditions. Surgery was successful at removing the lesion, and the patient was started on a 10-day dexamethasone taper and a 3-week levetiracetam course postoperatively (Table 1).

Three weeks postprocedure, the patient's mother reported the child had developed emotional dysregulation and “outbursts” uncharacteristic of her baseline functioning. The neurosurgery team noted that while transient mood fluctuations postoperatively are typical due to a myriad of factors (i.e. surgical trauma, medication side effects, and emotional burden of diagnosis), the nature and duration of her symptoms were uncommon. Postoperative MRI revealed nonspecific changes of fluid collection with increased T2/FLAIR signal along the resection cavity margin, with no tumor recurrence. Mother and child met with a social worker within the pediatric oncology clinic. In the subsequent 3 weeks, the patient reported the development of intrusive thoughts with fixation on stealing, leading to compulsive behaviors. She discarded all peer-given gifts, worried she had stolen them, and excessively questioned whether household items were stolen. She began apologizing excessively without reason to her mother and classmates. These symptoms intensified, causing social and emotional impairments, including struggling with school socialization and friendships, and displaying apathy toward others. She was referred to a child psychiatric clinic for further evaluation of her emotional and behavioral symptoms and supportive therapy.

A psychiatric evaluation was conducted using validated assessment tools and a clinical interview of parent and child. Initial completed measures at intake included:

• Child and Adolescent Symptom Inventory-5 (CASI-5), Parent: Met symptom count cutoff criteria for categories: D, generalized anxiety disorder (GAD); E, obsessions; F, social anxiety; H, schizoid personality disorder; K, major depressive disorder and persistent depression; M, Asperger's disorder.
• Screen for child anxiety related disorders (SCARED-Child): positive for GAD and social anxiety disorder.
• SCARED-Parent: positive for GAD.
• Child Depression Inventory 2 (CDI-2), parent report: score of 11, level of depression that is likely in the moderate range based on typical scoring.
• Child Depression Inventory 2 (CDI-2), child report: score of 2, low level of concern for depression.

Developmental history did not reveal exposures or delayed milestones. Complete family history was limited due to the inability to interview the biological father (child born following IVF with an anonymous male donor), however, the patient's biological mother reported that her family history was negative for psychosis or affective disorders. Mother had been diagnosed with anxiety in the past and treated successfully with sertraline. Child had no siblings and had resided with mother as primary caregiver her entire life, aside from a brief period of a few months during infancy when she lived with maternal grandmother. During the initial intake interview, the patient denied depression, anxiety, obsessive thoughts or compulsive behaviors, and psychotic symptoms. She acknowledged previous symptoms but stated these had resolved. Patient did endorse minimal anxiety about starting a new school and the risk of tumor recurrence. When discussing the incongruence between her current subjective report and the completed SCARED score, she explained that she had completed it based on her symptoms several weeks ago. Given the patient's denial of current OCD symptoms, additional validated tools, such as the Yale–Brown Obsessive–Compulsive Scale were not administered. Based on available data from objective measures and clinical interviews, she was tentatively diagnosed with unspecified anxiety disorder and agreed to continue to engage with the provider in weekly supportive therapy, in addition to ongoing monitoring of her symptoms.

Four months postprocedure, the patient began reporting consistent beliefs that loved ones, specifically friends and her dog, had been replaced by impostors, despite correctly recognizing their physical appearance. This manifested as identifying correct faces but experiencing associated unease and lack of familiarity. She described apathy towards friends and made comments to her mother that she suspected her dog “may have something wrong with his brain.” She reported that “I know that it's him on the outside, but there's something inside that's different”. She denied other false, fixed beliefs and did not report an obsessive nature to her thoughts or compulsive behaviors. The remaining psychiatric review of symptoms was also negative. Pediatric neurology consultation determined this misidentification and other emotional/behavioral symptoms were unlikely to be atypical seizures, with higher concern for tumor or postsurgical effects on the right frontal lobe. While a formal neuropsychological assessment was not available at the time, cognitive screening demonstrated normal executive function, recall, language, and visuo-spatial abilities.

After comprehensive evaluation, the diagnosis was documented as delusional misidentification syndrome, due to another medical condition. The patient began to engage in weekly psychodynamic psychotherapy with a child and adolescent psychiatrist, supplemented with cognitive-behavioral therapy techniques initially focused on false, fixed beliefs. Medications were considered but not initiated based on shared decision-making. Residual symptoms 6 months post-treatment included rare occurrences of obsessive thoughts and/or sudden mood changes (monthly), with complete resolution of false, fixed beliefs. Frequency of therapy sessions were decreased and at 1 year of treatment, she had complete resolution of her symptoms. Written informed consent was obtained from the patient's guardian for case report publication, and institutional review board determination of non-research was obtained.

## 3. Discussion

This case demonstrates a temporal relationship between frontal lobe tumor resection and the development of Capgras syndrome with associated obsessive–compulsive-like symptoms in an adolescent female. However, several alternative explanations must be considered beyond direct surgical causation, including postsurgical inflammation, emotional distress from hospitalization and diagnosis, medication effects, and potential predisposing neurobiological vulnerabilities.

Frontal cortical regions, including the prefrontal and orbitofrontal cortex, have been implicated in emotional regulation and executive function [1,2]. Disruptions in brain tissue from surgical intervention can lead to imbalances in neurotransmitter levels, including dopamine and serotonin. Dysfunction of dopaminergic and serotoninergic systems contributes to various psychiatric conditions, including mood disorders, anxiety disorders, and psychotic disorders [3].

### 3.1. Literature Review and Comparative Cases

Postsurgical psychiatric complications following right-sided frontal lobe resections remain limited in current literature. Mavros et al. [4] documented increased obsessionality following brain tumor surgery, particularly in female patients with left anterior hemisphere lesions, though right-sided presentations are less well documented. Several case reports describe similar postsurgical neuropsychiatric presentations.

Campanella et al. [5] reported acute personality and emotional changes following brain tumor resection, with symptoms typically emerging within weeks postoperatively and showing variable recovery patterns. Jenkins et al. [6] described emotional dysregulation and personality changes following frontal tumor resection, with younger patients showing different recovery trajectories compared to adults.

Transient Capgras syndrome has been reported following various neurosurgical procedures. Burns et al. [7] described a case of Capgras syndrome following right temporal lobectomy that resolved within 6 months. Silva et al. [8] reported misidentification syndromes following frontal lobe surgery with similar patterns of face recognition preservation but emotional familiarity disruption. However, the co-occurrence of both Capgras syndrome and obsessive–ompulsive-like symptoms in pediatric patients following right frontal resection remains exceptionally rare in existing literature.

### 3.2. Neuroimaging Considerations

The most consistent MRI findings in pediatric OCD include thalamic enlargement and gray matter alterations in frontal, insular, parietal, and temporal regions [9,10]. Radiological evidence of Capgras syndrome includes frontal subcortical white matter hyperintensities, temporal or occipital region hyperintensities, and cerebellar abnormalities [11,12]. One literature review demonstrated that 80% of abnormal imaging findings in Capgras cases were located in the right hemisphere, consistent with this patient's lesion location [13].

The absence of functional neuroimaging (fMRI and PET) in this case represents a significant limitation in understanding the underlying neural network disruptions. Functional imaging could have provided valuable insights into cortico-striato-thalamo-cortical (CSTC) circuit dysfunction associated with obsessive–compulsive symptoms and face-emotion processing networks implicated in Capgras syndrome, potentially informing treatment approaches and prognosis.

### 3.3. Limitations and Confounding Factors

Several limitations exist including the inability to establish definitive causation between frontal lobe tumor resection and the observed neuropsychiatric symptoms. Postsurgical inflammation may have affected limbic system and visual cortex areas responsible for facial recognition and emotional processing. Additionally, dexamethasone and levetiracetam have known psychiatric side effects that must be considered as contributing factors, though symptom persistence beyond medication cessation suggests other mechanisms. The incomplete family history due to anonymous sperm donation may have missed relevant predisposing neurobiological vulnerabilities.

## 4. Conclusion

This case underscores the importance of systematic neuropsychiatric screening and monitoring following right-sided frontal brain surgery in adolescents. While causality cannot be definitively established, the temporal relationship between surgery and symptom development warrants careful consideration of multiple contributing factors, including direct surgical effects, postsurgical inflammation, medication effects, and psychological trauma. Despite limitations, including a lack of functional neuroimaging, this case contributes to understanding complex interactions between neurological and psychiatric factors following frontal lobe pathology. Future research should incorporate pre and postoperative functional neuroimaging to better elucidate pathophysiological mechanisms and inform optimal treatment approaches.

## Figures and Tables

**Table 1:** Timeline of symptom progression.

Time post-surgery	Clinical findings	Interventions
3 Weeks	Emotional dysregulation and “outbursts;” intrusive thoughts about stealing; compulsive behaviors (discarding gifts, excessive questioning, apologizing), discontinues levetiracetam course	Social work consultation; repeat MRI shows expected surgical changes, reassurance provided
6 Weeks	Continued behavioral symptoms; social and emotional impairments, school difficulties	Referral to child psychiatric clinic
8 Weeks	Initial psychiatric evaluation; acknowledged past symptoms; minimized or denied current symptoms	Validated assessment tools administered; tentative diagnosis of unspecified anxiety disorder
4 Months	Development of Capgras Syndrome: beliefs that friends and dog were replaced by impostors	Pediatric neurology consultation; Comprehensive evaluation and diagnosis, weekly psychodynamic psychotherapy with CBT techniques initiated
4–6 Months	Gradual improvement in frequency and intensity of obsessive thoughts and delusional thinking/misidentification, and compulsive behaviors ceases	Medication was considered but not initiated; weekly psychodynamic psychotherapy with CBT techniques continues
6 Months	Rare occurrence of obsessive thoughts and mood changes (<1× monthly); complete resolution of misidentification beliefs	Therapy frequency decreased
1 Year	Complete resolution of all presenting symptoms	Discharge from psychiatric treatment

## Data Availability

Data sharing is not applicable to this article as no new data were created or analyzed in this study.

## References

[1] Campanella F., Fabbro F., Ius T., Shallice T., Skrap M. (2015). Acute Effects of Surgery on Emotion and Personality of Brain Tumor Patients: Surgery Impact, Histological Aspects, and Recovery. *Neuro-Oncology*.

[2] Jenkins L. M., Drummond K. J., Andrewes D. G. (2016). Emotional and Personality Changes Following Brain Tumour Resection. *Journal of Clinical Neuroscience*.

[3] American Psychiatric Association (2013). *Diagnostic and Statistical Manual of Mental Disorders*.

[4] Mainio A., Hä Hakko A. Niemelä, Salo J., Koivukangas J., Räsänen P. (2005). Level of Obsessionality Among Neurosurgical Patients With Brain Tumors. *The Journal of Neuropsychiatry and Clinical Neurosciences*.

[5] Campanella F., Mondani M., Skrap M., Vallesi A. (2019). Semantic Access Dysregulation in Non-Fluent Variant Primary Progressive Aphasia: Data From a Word Definition Task. *Neuropsychologia*.

[6] Jenkins L. M., Andrewes D. G., Nicholas C. L. (2014). Social Cognition in Patients Following Surgery to the Prefrontal Cortex. *Psychiatry Research: Neuroimaging*.

[7] Burns A., Jacoby R., Levy R. (1990). Psychiatric Phenomena in Alzheimer’s Disease. IV: Disorders of Behaviour. *British Journal of Psychiatry*.

[8] Silva A., Leong G. B., Weinstock R., Sharma K. K., Klein R. L. (1994). Delusional Misidentification Syndromes and Dangerousness. *Psychopathology*.

[9] Weeland C. J., White T., Vriend C. (2021). Brain Morphology Associated With Obsessive-Compulsive Symptoms in 2,551 Children From the General Population. *Journal of the American Academy of Child & Adolescent Psychiatry*.

[10] Huyser C., Veltman D. J., de Haan E., Boer F. (2009). Paediatric Obsessive-Compulsive Disorder, a Neurodevelopmental Disorder? Evidence From Neuroimaging. *Neuroscience & Biobehavioral Reviews*.

[11] Luca M., Patti A., Barbera N., Pirrone C., Carcione A. (2013). Clinical Features and Imaging Findings in a Case of Capgras Syndrome. *Neuropsychiatric Disease and Treatment*.

[12] Lewis S. W. (1987). Brain Imaging in a Case of Capgras’ Syndrome. *British Journal of Psychiatry*.

[13] Pandis C., Agrawal N., Poole N. (2019). Capgras’ Delusion: A Systematic Review of 255 published Cases. *Psychopathology*.

